# Influences of age, race, and sex on extracellular vesicle characteristics

**DOI:** 10.7150/thno.72676

**Published:** 2022-05-24

**Authors:** Nicole Noren Hooten, Anjali M. Byappanahalli, Mya Vannoy, Victor Omoniyi, Michele K. Evans

**Affiliations:** Laboratory of Epidemiology and Population Science, National Institute on Aging, National Institutes of Health, 251 Bayview Boulevard, Baltimore, MD 21224

**Keywords:** extracellular vesicles, exosomes, cargo, biomarker, age, race, sex

## Abstract

Recent attention has focused on the use of extracellular vesicles (EVs) as biological indicators of health and disease. These small, nano-sized membrane bound vesicles are secreted from cells into the extracellular space and can be readily isolated from bodily fluids. EVs can carry various bioactive molecules as cargo including DNA, RNA, proteins, and lipids. These EVs can provide a snapshot of the cell of origin and a window of opportunity to assess normal physiological states as well as pathophysiological states. For EVs to further develop as potential biomarkers of disease, it is important to characterize whether these vesicles and their associated cargo are altered in the context of demographic factors. Here, we summarize the current literature on how demographics such as age, race, and sex affect the levels and cargo of EVs. Age and sex influence both EV cargo and concentration while race studies report differences mostly in EV protein cargo. This review also identifies areas of future research and important considerations for the clinical use of EVs as biomarkers.

## Introduction

Extracellular vesicles (EVs) are small, nano-sized membrane bound vesicles that are released from cells into the extracellular space including body fluids. Early observational studies referred to EVs as “platelet dust or platelet particles” 1, 2, but decades of research have shown that these vesicles have a lipid bilayer and play important biological functions 3, 4. EVs are important mediators of intracellular communication between cells, tissues, and organs. Due to these roles, there is intense research on identifying if EVs can be used as biomarkers of disease 5, 6. Critical for biomarker development is the assessment of EVs in relation to demographic parameters including age, race, and sex. Here we summarize the status of the EV field across demographics and aim to identify different areas where more research is needed.

**Figure 1:**
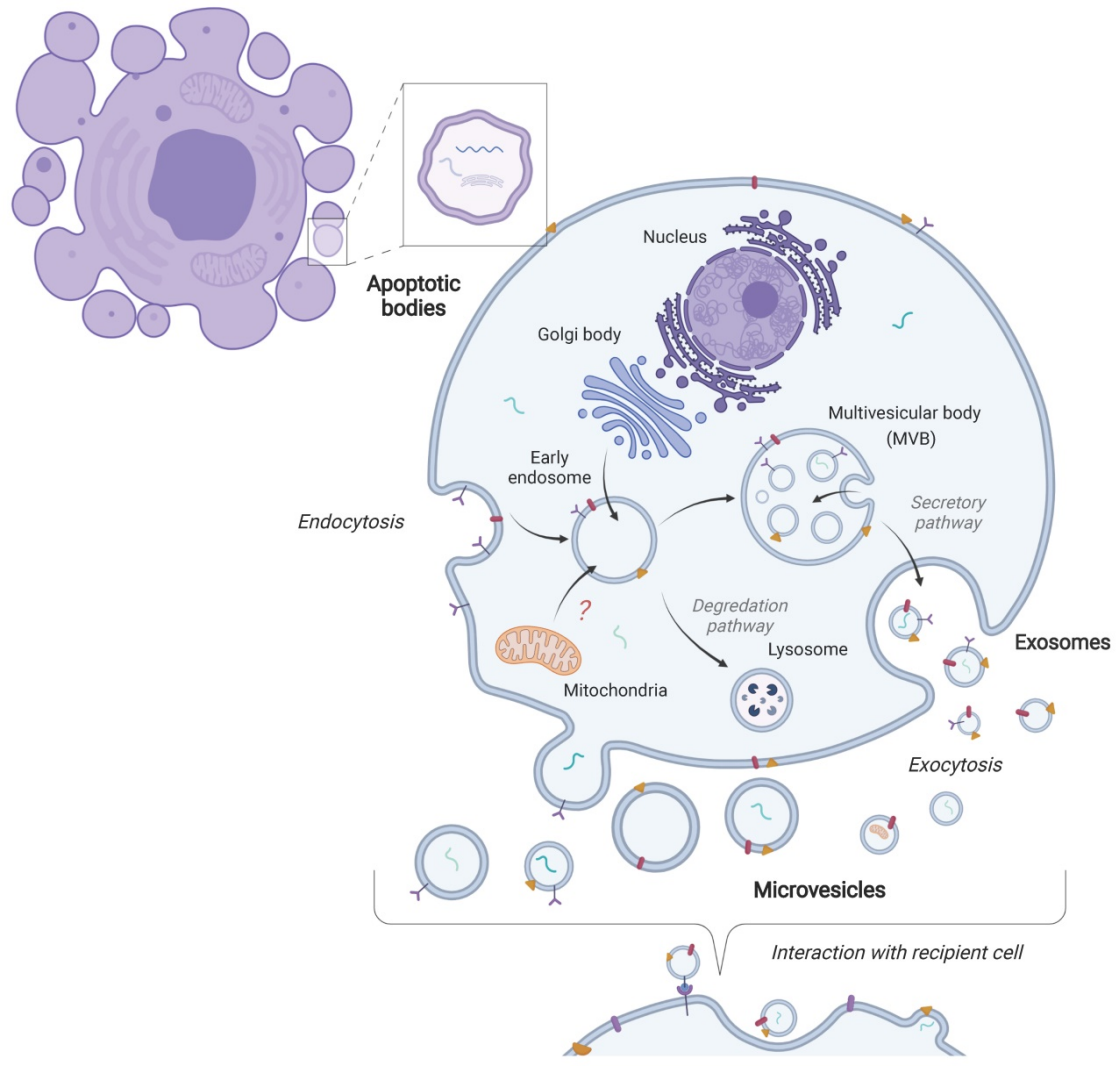
** Biogenesis of extracellular vesicles.** The three main classes of extracellular vesicles (EVs) differ in terms of their biogenesis. Exosomes are formed through the endocytic pathway and are released into the extracellular space upon fusion of the multivesicular body (MVB) with the plasma membrane. Microvesicles are formed through the budding of the plasma membrane. Apoptotic bodies are released as cell blebs during apoptosis. These vesicles then interact with recipient cells through various mechanisms.

EVs is a general term used to describe vesicles originating from several different biological processes 6. There are three main classifications of EVs in terms of their biogenesis: exosomes, microvesicles, and apoptotic bodies. Exosomes are contained within multivesicular bodies, which are created through the endocytosis of the plasma membrane. Multivesicular bodies then release EVs into the extracellular space through fusion with the plasma membrane 7-9. Microvesicles, on the other hand, are formed through budding from the plasma membrane 4, 10. Apoptotic bodies are formed through the process of apoptosis or cellular death 4, 10. Typically, EV size ranges are ~30-150 nm for exosomes, ~100-1000 for microvesicles and ~1000-5000 nm for apoptotic bodies 11. EVs can also be subtyped based on their size such as small EVs (< 100nm or < 200nm) and medium/large EVs (> 200nm) 12. A general schematic of EV biogenesis is shown in Figure 1. Determining the specific biogenesis pathway where EVs are derived remains difficult. In particular, EVs isolated from biofluids likely contain a heterogenous mix of EVs-derived from several processes and cell types. Therefore, here we will utilize the term EVs as this review focuses on EVs in biofluids where a particular biogenesis pathway cannot be designated (based on recommendations from the International Society of Extracellular Vesicles (ISEV)) 6, 12.

EVs contain nucleic acids (DNA, mRNAs, microRNAs (miRNAs), and other non-coding RNAs (ncRNAs)), lipids, and proteins; i.e., cytoskeletal, cytosolic, and plasma membrane proteins as well as vesicle trafficking proteins 4, 13-16. The concentration, contents, membrane composition, and size of EVs are highly diverse and depend on the cell of origin that released them. In addition, the cellular state influences EV cargo and concentration. Cargo can be used to characterize EVs and specifically protein composition can be analyzed to confirm the presence of EVs in preparations 12.

EVs can be isolated using different methods which include ultracentrifugation, density gradient centrifugation, size exclusion chromatography, and commercial precipitation reagents among many others 17, 18. Ultracentrifugation remains the most common method for EV separation, but other techniques are also gaining prominence 18. As the field evolves, technological advances and improvements have been made to the various techniques, yet there remain challenges in discriminating between different types of EVs. Furthermore, the different techniques all have various advantages and disadvantages, including purity, recovery, possible protein aggregates or contaminants, starting volume required, time, cost, and specialized equipment 17. Separating EVs in body fluids can be especially difficult due to their complex nature, where large amounts of proteins need to be separated out and input volumes are generally low. As a result, there should be special consideration of which technique to choose depending on the biological question and source of EVs.

## Functions of extracellular vesicles

EV cargo is functional and can be transferred to a recipient cell (Figure 1). Therefore, EVs can relay messages and information between cells and to distant tissues and organs. This ability makes EVs attractive for utilization as vehicles for therapeutics 19. In addition, EVs can bind to cell-surface receptors and elicit signaling cascades within the recipient cells. Thus, there is substantial interest in EVs since they can affect cellular function. Other functions have also been attributed to EVs including, but not limited to, the removal of cellular debris and trash, propagation of signals between tumor cells in the metastatic niche to the surrounding stromal environment, tumor vasculature, and immune systems 20-22. Through these various functions, EVs can elicit a multitude of biological pathways and responses. For example, EVs are important during immune responses, inflammatory processes, tissue repair, vasculogenesis, developmental processes, and stem cell differentiation 4, 23. Therefore, EVs are important for normal physiological processes but also can have roles in pathological processes. Accumulating evidence indicates key roles of EVs in many different conditions and diseases including cancer, diabetes mellitus and autoimmune, cardiovascular, and neurodegenerative diseases 4, 20, 24-27.

## Extracellular vesicles as biomarkers

Given the expansive roles of EVs in a variety of diseases, combined with the fact that EVs contain information related to cellular state, has led to immense interest in using EVs as biomarkers of various conditions and diseases. Furthermore, EVs are present and can be easily isolated from most all biological fluids such as plasma, urine, saliva, breast milk, synovial and seminal fluids, nasal and bronchial lavage fluid, cerebrospinal fluid, bile, uterine, and ascites 4. In particular, non-invasive blood-based biomarker development holds enormous promise as blood samples can be routinely taken and disease progression and monitoring of treatment regimens can be readily assessed. EV cargo including DNA, RNA, ncRNA (miRNAs and lncRNAs), and protein content are being explored as markers for disease (reviewed in 3). Approaches that combine analysis of multiple cargo types is also of consideration. Diseases affecting a variety of organ types are being evaluated, and future work lies in further developing strategies for using EVs as diagnostic and prognostic markers in disease.

Important for the clinical use of EVs is the assessment of EV characteristics and cargo in human populations. Furthermore, broadly characterizing EVs in diverse populations across ages and sex will enhance our understanding of whether EVs have clinical utility. This review explores the current status of EV research and reports the findings from the literature that examine EVs in the context of different demographics including age, race, and sex.

## Extracellular vesicles and age

### EV concentration with age

**Figure 2:**
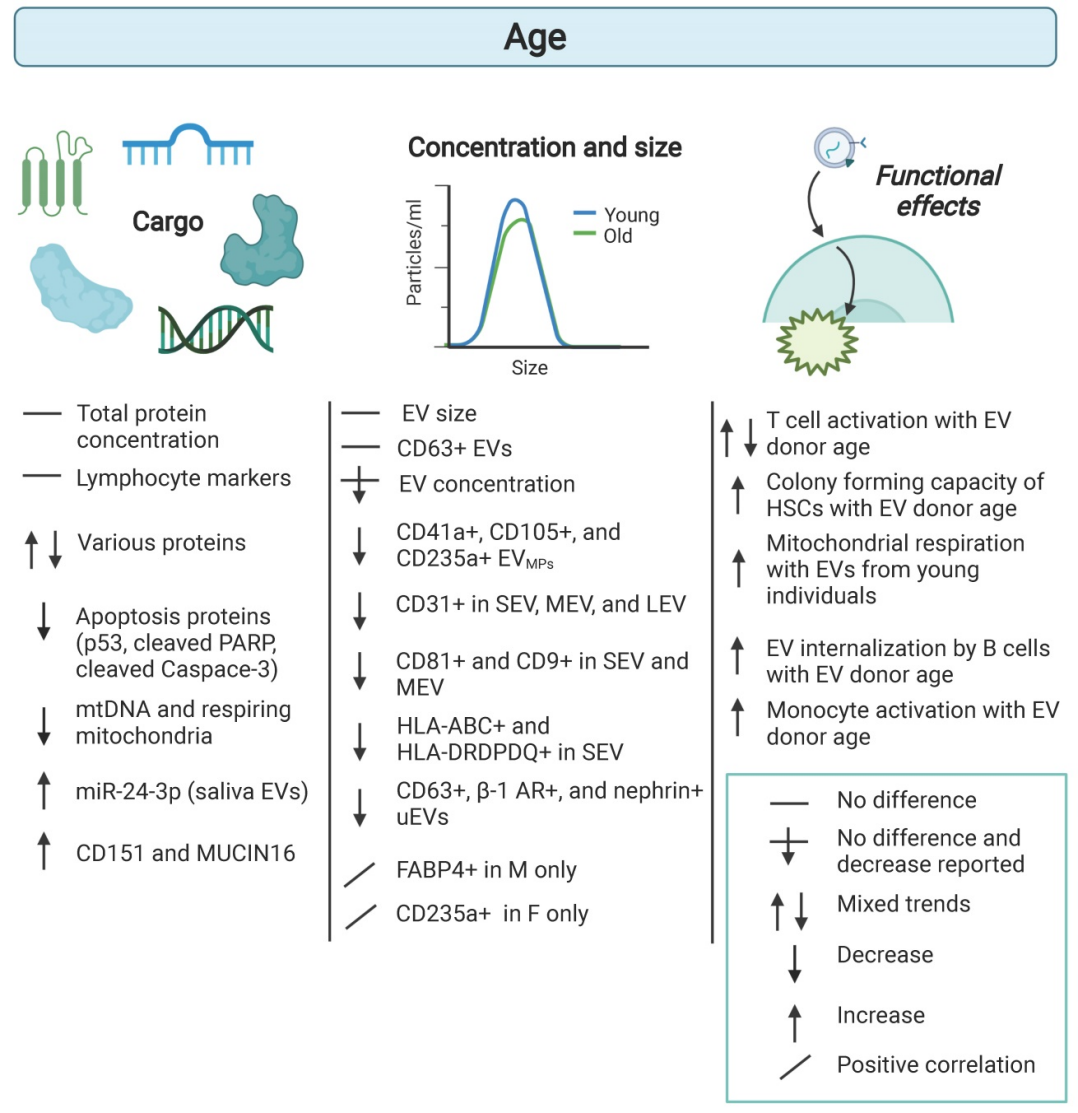
** Age-related differences in extracellular vesicles.** Human cohort studies have examined differences in extracellular vesicle (EV) cargo, concentration, and size in the context of age. EVs from different aged individuals elicit functional effects in recipient cells. Comparisons are indicated between old and young or with advancing age. Details are listed in Table 1. β-1 Adrenergic receptor (B-1 AR), small extracellular vesicles (SEV), medium extracellular vesicles (MEV), large extracellular vesicles (LEV).

The United States is approaching major demographic shifts in population. One demographic shift is the expected increase in the elderly population 28. By 2030, ~20% of the US population will be 65 and older 28. As this overall US population changes, so does the need to study individuals across the lifespan. Many EV studies do not incorporate age as a covariable or design cohorts with individuals from various ages. There is immense interest in identifying biomarkers of aging. These markers may reflect an association with chronological age or biological age, which is thought to indicate the rate that an individual ages. Gaining attention in the field is the utilization of circulating factors as indicators of aging and the aging process. Recent findings have implicated EVs as having a role in human aging 29. Currently model systems, most notably mouse models, have been employed to assess the role of EVs in aging (reviewed in 30, 31). However, here we will focus on the role of EVs in human aging. We have compiled EV research studies that have examined EVs with human aging in Table 1 and synthesized results from the field are shown schematically in Figure 2.

In a longitudinal study of aging, plasma samples were collected at two different time points approximately 5 years apart from a sub-cohort of African American and White adults who were young, middle-aged, and old from the Healthy Aging in Neighborhoods of Diversity across the Life Span (HANDLS) study. This approach allowed for both cross-sectional and longitudinal analysis of EVs in the context of age. Circulating EVs were isolated from these plasma samples and size and concentration were analyzed using nanoparticle tracking analysis. In both the cross-sectional and longitudinal analysis, EV concentration declined with advancing age 32. Exploring what may cause this decrease in EV concentration with age, EVs from individuals across the lifespan were incubated with freshly isolated human circulating cells, which include monocytes, T cells, and B cells. EVs from older individuals increased activation markers on monocytes compared to EVs from young individuals. Plasma EVs from older individuals were more readily internalized by B cells compared with EVs from younger individuals 32. This finding may explain, in part, the decrease in EV concentration in older individuals.

Cell-type specific EVs have also been identified in the circulation using standard flow cytometry for detection of EVs, which detects larger EVs traditionally referred to as microparticles ((MPs) and here referred to as EV_MP_) 27. To distinguish that these are larger EVs and to be consistent with terminology, we will use the term EV_MPs_ to refer to this population of EVs. In one study, EV_MPs_ positive for CD41a (platelet-derived), CD105 (endothelial-derived) and CD235a (erythrocyte-derived) were negatively associated with age. This cohort consisted of three age groups of ≤29 (n=32), 30-59 (n=61) and ≥60 years (n=15) 33. A more recent study used high resolution multicolor flow cytometry to characterize populations of circulating EVs by size and various cell surface markers 34. The percentage of different markers on EVs in the different size categories small EVs (SEV), medium EVs (MEV), and large EVs (LEV) were characterized in 12 healthy controls (age 40 ± 18 years) and 18 healthy controls (age 68 ± 8 years). In SEV and MEV subsets, the percentage of EVs with the EV markers CD81^+^ and CD9^+^ declined with age. The percentage of CD31, a surface marker for HSCs, T cells, B cells, NK cells, monocytes, macrophages and dendritic cells, positive EVs within all three subsets were significantly negatively associated with age 34. The percentage of the immunogenic molecules, HLA-ABC and HLA-DRDPDQ, on EVs declined with age in the SEV subset. These data suggested that specific populations of circulating EVs may also be altered with human age.

It should be noted that lower EV levels in older individuals have not been observed in all studies 35, 36. Other studies have shown no differences in EV concentration with advancing age 35, 36. These differences may lie in lower cohort numbers and different age ranges in these cross-sectional studies. The study by Hajian et al. comprised young males (n=3; age range of 25-35 years) and old males (n = 3; age range of 65-75 years). In the Grenier-Pleau et al. study, individuals were young (n=12; 20-39 years), middle-aged (n=11; 40-59 years), and older (n=12; 60-85 years). The aging cohort in Eitan et al. were young (n=30; 30-35yrs), middle-aged (n=30; 40-55 yrs;), and old (n=14; 55-64yrs) at time 1. As this was a longitudinal cohort, 74 individuals were assessed at both time 1 and at time 2 (n=148). In addition, different techniques were used to separate EVs in these studies. Interestingly, in a recent mouse study, a decrease in circulating EV concentration with age was reported using several different EV isolation methods 37.

Another major emerging field of EVs is the study of urinary EVs (uEVs). Turco et al. examined age-related differences in EVs isolated from urine using flow cytometry 38. With advancing age, there was a decrease in the total number of CD63^+^, juxtaglomerular cell (β-1 adrenergic receptor^+^) and podocyte (nephrin^+^) markers on uEVs 38. As examining uEVs with age is an emerging topic, we certainly have much to learn about age-dependent changes in the composition and amounts of these EVs from urine.

### Differences in EV cargo with age

Changes in EV cargo with human age has also been described. Apoptosis markers including p53, cleaved PARP, and cleaved Caspase-3 were decreased with age in human plasma EVs 32. EV associated CD151 has been shown to be higher in aged individuals in multiples studies 32, 36. Differential EV protein cargo with age has also been implicated in the ability of EVs from older individuals to stimulate hematopoietic stem cells 36. Activation of monocytes or T cells is also affected by the age of the EV donor 32, 39. These data suggest that EVs from aged individuals may affect cellular function differently. Consistent with this idea, EVs from aged donors impaired cellular mitochondrial respiration compared to EVs from young donors 40. In this study, different molecular cargo was attributed to these cellular effects. This study used plasma EVs from a previous study 32 and found that plasma EVs contain circulating cell-free mtDNA (ccf-mtDNA) 40. Furthermore, EV mtDNA levels declined with human age 40. Consistent with these findings, active mitochondria have been detected in circulating EVs with MitoTracker™ Deep Red FM 34. In this study, it was reported that the percentage and numbers of CD29^+^ and CD31^+^ EVs with active mitochondria decreased with advancing age 34. The percentage of CD9^+^ EVs carrying respiring mitochondria also declined with human age. The mean fluorescent intensity of MitoTracker™ Deep Red decreased with age in multiple EV subpopulations 34. These data point to mitochondrial components as EV cargo that may be important during the aging process 41. In fact, mitochondrial components have recently gained interest as EV cargo 41, 42.

Other types of EV cargo have also been reported to be altered with human age. For example, emerging evidence suggests that miRNA levels may be altered in EVs with age 43, 44. miRNAs are short ncRNAs that regulate gene expression post-transcriptionally. These ncRNAs are abundant and are very stable in plasma/serum 43. This stability is thought to be due to miRNAs being bound to lipoproteins, RNA-binding proteins or encapsulated in EVs. Thus far, most studies examining miRNAs with human aging isolate miRNAs from plasma/serum and do not differentiate whether the miRNAs are in EVs 43, 45. In addition, the majority of the findings are in mice 43, 45.

Data suggests that miRNAs are important mediators of the aging process 46, 47. A pilot study found that salivary levels of EV associated miR-24-3p were higher in aged individuals 48. A subset of miRNAs have been termed “inflammamiRs” as they appear to have key roles in stimulating the low-grade, chronic inflammation observed in the elderly termed “inflammaging” 49, 50. Several of these miRNAs, including miR-146 and miR-21, can be encapsulated in EVs and may impact aging by modulating inflammation through regulating the NFκB pathway 44. In addition to inflammatory pathways, these miRNAs are predicted to target other pathways important for aging including DNA damage response, senescence, oxidative stress, and proteostasis 43, 45. As miRNAs contained in EVs can be transferred and functional in recipient cells and a single miRNA can have multiple targets, these data point to miRNAs as being important EV cargo in aging. In addition, other RNA biotypes, including long noncoding RNAs, circular RNAs and mRNAs, also are contained in EVs and have been associated with aging or age-related diseases 14. Ongoing efforts will surely expand upon these studies to elucidate both the coding and noncoding RNAs that function in EVs during aging. Thus far, most studies focus on these miRNAs in the context of age-related diseases such as type 2 diabetes, cardiovascular diseases, and neurodegenerative disorders or in mice and do not separate miRNAs from the EV fraction, but rather examine miRNAs in whole plasma/serum (reviewed in 43, 45). Future work lies in deciphering further the EV encapsulated versus circulating pools of RNAs and their specific roles in the aging process.

Although not the focus of this review, it should be mentioned that EVs from young mice have been utilized as therapeutics to dampen age-related disease and lengthen lifespan in mice 30. These data build upon decades of research using parabiosis treatment in mice. Circulation from a young mouse can have beneficial health effects in the old mouse 30. The seminal work by Yoshida and colleagues provided experimental evidence that injection of EVs from young mice into old mice increases mouse lifespan 51. Follow up studies are warranted but this mouse data points to EVs as important circulating mediators that could explain the benefits of a young circulation on an older mouse in the parabiosis paradigm.

## Extracellular vesicles and race

**Figure 3:**
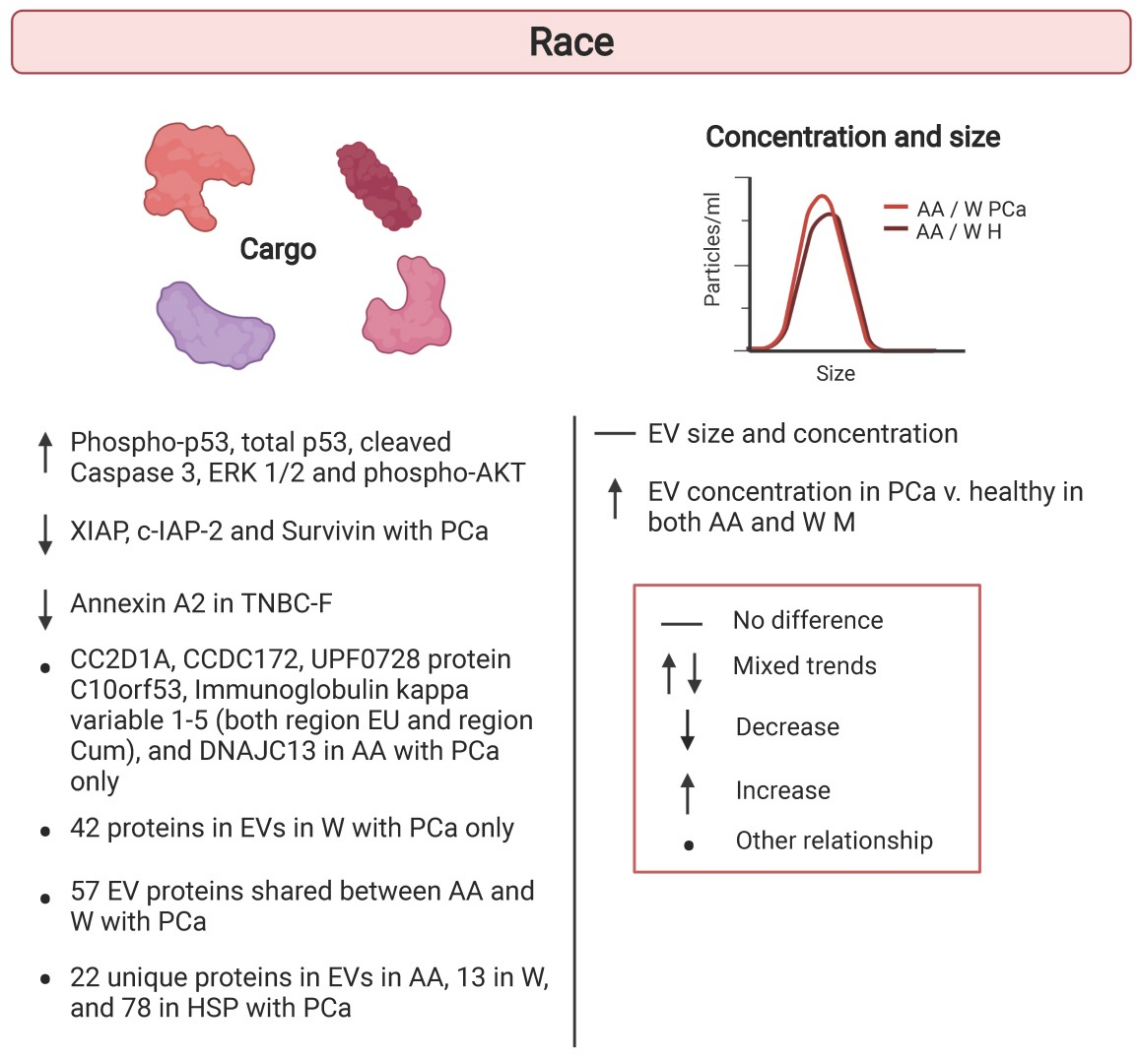
** Racial differences in extracellular vesicles.** Human cohort studies have examined differences in extracellular vesicle (EV) cargo, concentration, and size in the context of race. Comparisons are indicated between White versus African American individuals. Details are listed in Table 2. African American (AA); Healthy (H) Hispanic (HSP); Prostate Cancer (PCa); Triple negative breast cancer (TNBC); White (W).

Another major demographic shift in population is approaching in the US. It is estimated that by 2060, 56.4% of the U.S. population will be composed of individuals belonging to a minority group 52. This shift indicates that in 2060 non-Hispanic Whites will no longer be the majority population in the US. As these changes in demographics occur, studies should reflect these shifts and incorporate racially and ethnically diverse populations. Thus far, few studies have considered the role race might play in altering EV characteristics (Table 2). Therefore, understanding how race would alter EV size, concentration, and protein cargo should be a priority for such investigations to fully understand EVs within the population. Here we have highlighted the studies examining EVs in the context of race and ethnicity and these studies are listed in Table 2 and shown schematically in Figure 3.

In addition to changes in population demographics, it is important to incorporate populations experiencing health disparities into research studies. Understanding the root causes of disparities will aid in prevention and development of therapies targeted to at-risk populations. For example, in the US, disparities in mortality rates persist among racial groups. African American individuals have a higher risk for mortality compared to Whites individuals 53-56. Therefore, there is an unmet need to identify molecular markers that may indicate individuals at risk for early mortality in racially diverse cohorts. Little is known about whether EVs can possibly be used as biomarkers for mortality. It is necessary to examine the association of EV characteristics with existing well-established clinical markers of mortality.

To do this, EVs were explored in the context of mortality using plasma samples from a sub-cohort of African American and White adults from the HANDLS longitudinal study 57, 58. The benefits of utilizing samples from participants in a longitudinal clinical study is the availability of a large database of existing clinical data. Therefore, the association between plasma EV size, concentration, and protein cargo with clinical measurements that previously have been associated with mortality were examined. EV characteristics, specifically EV protein cargo, were found to vary between African American and White participants. African American adults in this study had lower levels of phospho-AKT, phospho-p53, total p53, cleaved Caspase-3 and ERK1/2 in EVs 58. Many EV proteins and clinical mortality markers differed in their relationship by race. For example, markers of renal function including creatinine and estimated glomerular filtration rate (eGFR) showed a significant race interaction with several EV proteins. The association of metabolic markers such as homeostatic model assessment (HOMA) of β-cell function (HOMA-B) and other clinical markers of mortality, cholesterol, mean cell volume (MCV), serum alkaline phosphatase (ALP), lactate dehydrogenase (LDH), and mean arterial pressure (MAP) with various EV protein levels differed by race 58. Other EV characteristics including EV concentration and size did not show significant changes with race. This is consistent with previous findings in an aging cohort 32. EV concentration was significantly associated with several markers of mortality and there was an interaction with race for cholesterol 58. EV concentration and levels of insulin signaling, apoptosis, and inflammatory proteins do vary with different mortality risk factors and their relationships differ with certain factors between African American and White adults. Race is an important aspect to consider, shown in these studies to be an important social determinant in EV cargo differences, as many EV studies strive to adopt EVs as clinical biomarkers of a variety of diseases.

### Racial differences in EV cargo in cancer

Racial differences in EV cargo have also been identified in prostate cancer, but this work is prelimnary. This is an important avenue of exploration as there are large racial disparities in prostate cancer. For example, African American males suffer higher incidence, worse prognosis and increased mortality compared to White males 59. Therefore, it is important to identify the molecular determinants that may underly these prostate cancer disparities.

Both African American and White males with prostate cancer were reported to have higher circulating levels of EVs compared to healthy males 60. In both races, EV size was consistent between cancer and healthy participants 60. EV proteomic analysis revealed that African American and White prostate cancer patients shared about 57 proteins, but there were 6 proteins found exclusively in EVs from African American patients and 42 found exclusively in White patients with prostate cancer. Another study also examined proteins that were different in EVs from African American and White males with prostate cancer. In this study, inhibitors of apoptosis proteins (IAPs), a known family of oncoproteins, were higher in plasma EVs from African American patients with prostate cancer compared to White patients 61. In another small study, proteins in serum EVs were identified that either overlapped or were unique in African American, White, and Hispanic prostate cancer patients 62. Given the health disparities in prostate cancer, this avenue of research holds promise and larger scale studies are warranted to validate these initial findings.

In females with triple-negative breast cancer (TNBC), levels of annexin A2, were elevated in plasma EVs from African American females compared to White females 63. African American females are more likely to suffer from this aggressive subtype of breast cancer and TNBC has very little targeted treatment options 59. Therefore, it is important to gain a better understanding of potential prognostic indicators in TNBC.

We are only beginning to assess the racial differences in EV characteristics and cargo. These studies point to alterations in EV protein cargo in African American versus White individuals (Table 2; Figure 3). Larger studies are needed to validate these findings. Additionally, more expansive investigation of various EV cargo, such as RNAs, and EV concentration, and size between racial and ethnic groups is needed to improve clinical use of EVs in diagnostics of disease.

## Extracellular vesicles and sex

**Figure 4:**
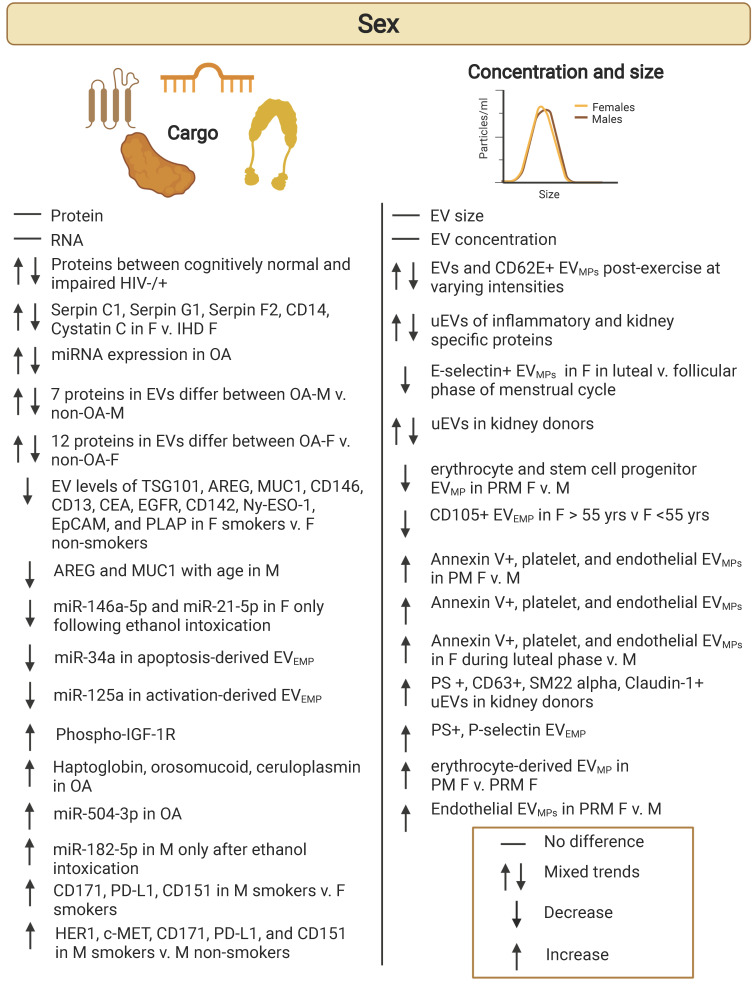
** Sex differences in extracellular vesicles.** Human cohort studies have examined differences in extracellular vesicle (EV) cargo, concentration, and size in the context of sex. Comparisons are indicated between females versus males unless stated otherwise. Details are listed in Table 3. Females (F); Ischemic heart disease (IHD); Males (M); Osteoarthritis (OA); Post-menopause (PM); Pre-menopause (PRM).

Several human studies have explored if there are sex differences regarding EV cargo, concentration, and size (Table 3; Figure 4). In these studies, EVs were isolated from a variety of bodily fluids including plasma 33, 58, 64-69, urine 38, 70, 71, and synovial fluid 72, 73. Sex differences have also been explored in EVs from brain and skeletal muscle tissues 74, 75. These characterizations of EVs have been explored in various pathologies and conditions such as cardiomyopathy 76, osteoarthritis 72, 73, neuroinflammation 67, stable ischemic heart disease (IHD) 65, obesity 77, HIV 69, and kidney stones 70. EVs have also been studied in the context of various lifestyle behaviors including acute exercise states and exercise intensity 68, 75, smoking 33, 64 and nicotine addiction 74. Understanding sex-specific differences in EVs is important as we move towards utilizing EVs in diagnostic and therapeutic approaches. Many diseases affect males and females differently 78. Yet, this variable is often overlooked, especially regarding EV studies. There is an important need for sex-specific studies that incorporate both sexes. Sex refers to the biological component and gender refers to the socially constructed characteristics. As most of the studies reviewed here report sex differences, we will be referring to sex rather than gender throughout.

### Differences in EV size and concentration by sex

Some studies have characterized EV concentration and size by sex. One study by Enjeti et al. characterized circulating EVs in healthy subjects 33. Enjeti and colleagues found that the median levels of CD41, CD235, TF (tissue factor), and PS (phosphatidyl serine) expressing EV_MPs_ were generally similar in females compared to males. Effects of various stimuli and conditions have also been explored regarding EV concentration and size regarding sex. Recent reports also suggest that exercise influences circulating EV populations in males and females. Rigamonti et al. explored the effect of acute exercise on EVs 79. They found that levels of exosome (30-130 nm) and microvesicle (130-700 nm) populations of EVs were lower and higher in females compared to males post-exercise, respectively. Another study looked at the effect of exercise intensity (moderate vs. high) on EVs 68. It was reported that compared to pre-exercise, moderate-intensity continuous exercise lowered CD62E^+^ EV_MP_ populations post exercise in females, but not in males. High-intensity interval exercise had no effect on CD62E^+^ EV_MP_ populations. In males, elevated CD62E^+^ concentrations were observed after high-intensity interval exercise compared to moderate-intensity continuous exercise.

Sex-specific differences in EV concentration from urine have also been described. For example, a recent article reported that females excrete fewer uEVs than males 71. However, this contrasts with a previous article that found more uEVs were excreted from females than males 38. Different EV techniques were used to quantify uEVs in these studies: ExoQuant 71 and flow cytometry 38. In addition, different strategies for adjusting to creatinine levels were also utilized. Blijdorp and colleagues also measured total kidney volume, which was lower in females than males and the authors suggest that nephron mass should also be taken into account. Turco et al., also examined uEVs positive for various cell-type specific markers and reported that the median levels of PS^+^, CD63^+^, mesangial cell (SM22 alpha^+^) and Bowman's capsule-parietal cell (claudin-1^+^) markers were higher in females compared males. Future work lies in further characterizing how sex may influence the levels and various cell-type specificity of uEVs.

Differences in uEVs by sex have also been studied in the context of kidney stones 70. As males have nearly two times the incidence of kidney stones compared to females, EVs may give clues to the mechanisms that may cause this disparity. In a study conducted by Jayachandran et al., uEVs from 110 persons who presented with their first kidney stone incident were isolated from urine samples (n = 50 females, 60 males) with ages ranging from 19-76 years of age 70. uEV_MPs_ were characterized using flow cytometry to determine concentration of uEV_MPs_ depending on origin from specific nephron segments (transitional epithelium, podocytes, parietal cells, simple cuboidal epithelium, simple squamous epithelium, or principal/intercalated cells). Levels of EV positive biomarkers (phosphatidylserine and CD63) were assessed, as well as cell adhesion and inflammatory proteins (E-cadherin, ICAM-1, VCAM-1, tissue factor, and MCP-1).

Comparisons were made between age-matched, kidney stone status groups. Compared to control males, control females had higher levels of uEV_MPs_ positive for PS, CD63, VCAM-1, TF, MCP-1, podocin plus galectin-1, claudin-1, URAT1, SLC14A2, uromodulin, SLC12A3, aquaporin-2, V-ATPase, cytokeratin 19, and neprilysin. Comparison between control females to females with kidney stones showed that levels of CD63 positive uEV_MPs_ were greater in females with kidney stones compared to control females. In contrast, levels of uEV_MPs_ positive for VCAM1, podocin plus galectin-1, claudin-1, URAT1, SLC14A2, uromodulin, SCL12A3, V-ATPase, cytokeratin 19, and neprilysin were all lowered in females with kidney stones compared to control females. This shows that levels of uEV_MPs_ from the studied nephron segments were all lowered in females with kidney stones compared to control females. Similar comparisons between control males to males with kidney stones revealed that levels of phosphatidylserine and CD63 were higher in males with kidney stones. Sex-based comparisons showed that levels of CD63, MCP-1, and podocin plus galectin-1 positive uEV_MPs_ were higher in females with kidney stones compared to males with kidney stones. Overall, there was a greater number of uEV_MPs_ from control females compared to control males and kidney stone-forming males and females. This study shows the importance of characterizing levels of uEVs from various cell-specific origins in both the absence and presence of the disease pathology of interest.

The role of hormones and menopause in females has also been studied on EV populations. Results from a 2017 study on circulating EV number, function, and content by biological factors, such as sex, reported lower numbers of EV_MP_ associated CD105 in females above the age of 55 years 33. A similar study on a cohort of relatively healthy individuals reported that EVs from erythrocytes (CD235a^+^) and stem cell/progenitor cells (CD117^+^) were significantly lower in pre-menopausal females than age-matched males 66. However, endothelial-derived EVs (CD62E^+^) were higher in pre-menopausal females compared to age-matched males. They also reported that erythrocyte-derived (CD235a^+^) EV_MPs_ were higher in post-menopausal females compared to premenopausal females 66. The levels of erythrocyte-derived EVs (CD235a^+^) were correlated with age in females but not males, whereas adipocyte-derived EVs (fatty acid binding protein/FABP4^+^) were correlated with age in males but not females. Interestingly, overall females in this cohort had higher levels of PS^+^ and P-selectin^+^ EV_MPs_ compared to males.

This link to a hormonal influence on circulating EVs has been extensively explored in another recent study 80. In a cohort of 27 healthy females, the role of different phases of the menstrual cycle was compared to age-matched males (n = 18). In contrast to age-matched males, females had higher levels of circulating annexin V^+^ EV_MPs_, and EV_MPs_ derived from platelets (CD61^+^ and P-selection^+^) and endothelial (E-selectin^+^) cells. During the luteal phase of the menstrual cycle, females had elevated levels of EV_MPs_, including platelet-derived EVs (CD61^+^, P-selectin^+^ and CD63^+^) as well as endothelial cell-derived EVs (E-selectin^+^) compared to males. Except for E-selectin positive EVs, all the other populations were higher in females in the luteal phase versus follicular phase of the menstrual cycle 80. Therefore, not only were sex differences observed in baseline levels of circulating EV_MPs_ populations, but these effects were modulated by the menstrual cycle.

While there are few studies that have examined how sex influences EV concentration and size in humans (Table 3; Figure 4), these characteristics must be fully understood to better understand the role of EVs in the normal physiology and in maintaining homeostasis as well as in pathological conditions.

### Differences in EV cargo by sex in healthy individuals

There have been studies that have explored sex-differences in EV cargo in healthy individuals. Most of the studies on EV cargo surround exploration of various proteins and miRNAs of interest. A few studies have shown that there are differences in EV cargo between males and females 33, 58, 81. One study from 2017 conducted by Bammert et al. explored EV associated miRNA content 81. This study compared plasma-derived subpopulations of EVs and their miRNA cargo between males and females. Circulating EV_MPs_ were quantified using flow cytometry and endothelial-derived EVs (EV_EMPs_) were sorted based on markers indicative of activation (CD62E^+^) or apoptosis (CD31^+^/CD42b^-^). There were no differences in circulating concentrations of activation- and apoptosis-derived EV_EMPs_; however, examination of the miRNA cargo revealed that there were differences in EV cargo from activation-derived EVs between males and females 81. Most notably, miR-125a levels in activation-derived EV_EMPs_ were lower in males and miR-34a levels in apoptosis-derived EV_EMPs_ were higher in males compared to females.

Plasma EV proteins have also been analyzed. From a cross-sectional cohort of middle-aged African American and White individuals, it was reported that phospho-IGF-1R levels in EVs were significantly higher in females compared to males 58. No sex differences were reported in EV concentration, size distribution, mean EV size, EV size mode or other EV associated proteins related to insulin signaling, apoptosis proteins, and other signaling proteins.

Another study also used flow cytometry to analyze sex differences in EV_MPs_ in a cohort of 143 (males = 80, females = 63) healthy individuals 33. There were no significant sex differences in the surface levels of CD41, CD105, CD235, TF and PS on EV_MP._ Coagulation activity was measured in the EV_MPs_ and it was found that females had higher levels of procoagulant activity levels. In a different cohort of individuals (n=35), RNA was isolated from EV_MPs_ and total amounts of small RNA and miRNAs were measured. Although no differences were reported, these findings should be verified by RT-qPCR rather than relying on Agilent Bioanalyzer quantification for total quantities, as total amounts may not vary but individual miRNAs may vary.

Thus far, very few studies have explored sex-differences in EVs and their associated cargo. Additionally, studies reported are in a narrow focus with interest in a select population of EV cargo. More research is required to characterize if there are differences in individuals in the absence of disease, so that they may be compared to varying disease states or other biological factors that may affect EV cargo. As stated previously, mainly EV protein and miRNAs have been studied; however, EVs can carry several other types of cargo, including but not limited to DNA and lipids. Further research into these cargos with respect to sex-differences opens an even larger potential for EV-based diagnostic and therapeutic tools. Characterization of these subsets should also be taken into consideration when exploring EV cargo for biomarkers of health. It is critical to consider sex-dependent differences when studying EV cargo in other disease states. The development of EV-based diagnostics requires us to understand these baseline levels.

### Sex differences in EV cargo with clinical conditions

Sex-differences in EV cargo in the context of various diseases and conditions have also been described. Most of the differences found in EV cargo reside in differential up-regulation and down-regulation of proteins and various miRNAs. Using an EV array, Baek and colleagues examined plasma EV profiles in a cohort of 161 plasma samples from healthy individuals 64. While they found that there were no differences observed in the EV cargo between males and females, a differential profile was seen when including smoking status. EVs from male smokers had higher levels of CD171 (L2CAM), PD-L1 (programming cell death ligand 1), and TSG101 (tumor susceptibility gene 101) compared to female smokers. EV levels of TSG101, AREG (amphiregulin), MUC1 (CA15-3), CD146 (MUC18), the alanine aminopeptidase CD13, CEA (carcinoembryonic antigen), EGFR (epidermal growth factor receptor), CD142 (coagulation factor III, tissue factor), Ny-ESO-1 (New York esophageal squamous cell carcinoma-1), EpCAM (epithelial cell adhesion molecule), and PLAP (placental alkaline phosphatase) were significantly lower in female smokers compared to female non-smokers. HER2 (human epidermal growth factor receptor 2), c-MET (mesenchymal-epithelial transition factor), CD171, PD-L1, and CD151 levels were higher in male smokers compared to male non-smokers. In addition, males have lower EV amounts of AREG and MUC1 with advanced age. Therefore, smoking may cause cellular changes that may have different consequences on EV protein profiles in females versus males.

Another study compared EV cargo between males and females with and without osteoarthritis (OA) 72. The authors showed that miRNAs in EVs isolated from synovial fluid are altered in OA and that there were differences in EV miRNAs between males and females in these comparisons. Most notably, 69 synovial EV miRNAs were significantly downregulated in males with OA compared to non-osteoarthritic (non-OA) males. In females with OA, 91 synovial EV miRNAs were downregulated and 52 were upregulated compared to non-OA females. In a follow-up study, the authors characterized synovial EV proteins in males and females with and without OA. In females with OA, it was reported that haptoglobin, orosomucoid, and ceruloplasmin were significantly higher while apolipoprotein was lower in synovial EVs compared to non-OA females 73. In males with OA, β-2-glycoprotein and complement component 5 proteins were significantly elevated and Spt-Ada-Gcn5 acetyltransferase (SAGA)-associated factor 29 was lower in synovial EVs compared to non-OA males 73. These results indicate that miRNAs and protein cargo in synovial EVs are altered in males and females with osteoarthritis.

Sex differences have also been reported in EV cargo proteins in ischemic heart disease (IHD). In a study conducted by Dekker and colleagues, EVs were isolated from the plasma of patients with stable IHD (n=187) and patients without IHD (n=257) as part of the MYOMARKER (MYOcardial ischaemia detection by circulating bioMARKERS) study 65. Proteins were quantified from 3 different EV-containing lipid fractions from plasma. Six proteins were quantified from these plasma fractions including serpin C1, serpin G1, serpin F2, plasminogen, CD14, and cystatin C. Results showed that there was a difference between several proteins in plasma EV fractions in patients with and without stress induced IHD. More interestingly, sub-group analysis based on sex demonstrated these associations were only seen in females with and without stress induced IHD. This study highlights the importance of examining sex-specific EV cargo changes.

Neuroinflammation also appears to affect EV cargo in a sex-specific manner. One study analyzed ethanol intoxication as a model for the role of neuroinflammation on EV cargo in mice and humans 67. The study included adolescent and young adults (n=18) who were admitted to the emergency department due to moderate to severe alcohol intoxication, in addition to 18 healthy controls (males = 9, females = 9). Following ethanol intoxication, both human and mouse females had lower levels of the anti-inflammatory miRNAs, miR-146a-5p, miR-21-5p, miR-182-5p, in plasma EVs, while human and mouse males had higher levels of the same miRNAs after ethanol intoxication.

Sex-specific EV cargo protein differences have also been studied in the context of HIV 69. Plasma neuron-derived EVs (NDEs) were isolated from a cohort of 80 individuals, including 51 females and 29 males. There were 4 comparison groups, each consisting of 20 individuals: HIV-negative neuropsychologically normal (NPN), HIV-positive NPN, HIV-positive asymptomatic neurocognitive impairment (ANI), and HIV-positive mild neurocognitive disorder (MND). Levels of proteins that play a role in neurocognitive diseases were analyzed in NDEs.

When analyzing levels of known markers for neuronal damage in NDEs, levels of high mobility group box 1 (HMGB1), neurofilament light (NfL), p-T181-tau, and amyloid beta (Aβ) were compared across the control group (HIV-negative NPN), HIV-positive NPN, and combined HIV-positive asymptomatic neurological impairment (ANI) and mild neurocognitive disorder (MND) 69. NDE cargo from HIV-positive ANI and MND males had increased levels of HMGB1 and NfL compared to levels in NDEs from the control and HIV-positive NPN groups. HIV-positive ANI and MND females did not have significantly different levels of HMGB1 compared to NDEs from control and HIV-positive NPN groups, but HIV-positive NPN females had higher levels of NfL compared to control and combined HIV-positive ANI and MND females. HIV-positive NPN males and combined HIV-positive with ANI and MND males had significantly lower levels of NDE p-T181-tau compared to control groups, while there was no difference amongst females. There were also no significant differences in Aβ NDE levels between the male or female groups.

Proximity extension assays (PEA) were used to compare three HIV-positive groups between males and females: NPN, ANI, and MND (n = 34 for females, n = 22 for males). The analysis showed that there were 7 NDE proteins in HIV-positive females with cognitive impairment and 12 NDE proteins in HIV-positive males with cognitive impairment that were significantly different compared to NPN groups. These results suggest sex differences in NDE protein content with HIV infection and show promise for using these proteins for future diagnostic purposes for HIV-associated neurocognitive disorders (HAND), although this cohort was relatively small, thus the results should be replicated in larger studies.

These data suggest that various lifestyle factors and disease processes affect EV cargo differently between the sexes. This indicates that EVs should be furthered characterized in many other diseases and conditions. As there are few studies in this area, future extensive research should be directed at examining baseline levels of EV cargo of interest between males and females.

In addition, cohorts should be extensively described, and covariates should be incorporated into the statistical analyses. It is difficult in many EV studies to decipher the cohort characteristics, statistical methodologies used, and the lifestyle and clinical variables of the cohort. Age, race, and sex should all be reported. Factors such as smoking, dyslipidemia, hypertension, diabetes, obesity, and large- or small-artery atherosclerosis (including coronary heart disease, stroke, and peripheral artery occlusive disease) might play a role in explaining age, race, or sex differences in EV cargo, yet many of these variables are not incorporated into statistical analyses in EV studies. For example, for the papers we reviewed here only Machida et al. 48, Eitan et al. 32, Enjeti et al. 33, Sun et al. 69, Noren Hooten et al. 58, Dekker et al. 65, Rigamonti et al. 79, and Lazo et al. 40 included covariates into the statistical analyses. Therefore, it is important for future EV studies to include appropriate covariates in statistical analyses. In addition, many “clean cohorts” focus on particular populations and many cohorts are not “cleanly phenotyped”. The EV field is evolving at a rapid pace with growing technological improvements for characterizing EVs. Thus far, efforts on promoting rigor and standardization in the field have focused mostly on the characterization of EVs 12, 82, 83. Therefore, it will be important in the future to improve upon reporting, statistical methodologies, and incorporating clinical characteristics of cohorts into EV studies.

## Conclusions and Future Perspectives

The field of EVs has grown exponentially, in part due to their vast biological roles but also because of their potential ability to be used as diagnostic and prognostic factors for diseases and conditions. In fact, >500 patents were filed in the US for the utilization of EVs from 2000-2020 84. This fact highlights the potential clinical and commercial use of EVs. Furthermore, this expanded interest is also reflected in the growing numbers of registered clinical trials that utilize EVs 19. The membrane-bound structure of EVs also makes them a potential as drug delivery tools that could decrease undesirable, immunogenic responses. Although there is substantial and growing interest in EVs, we still lack a universal understanding of EVs in non-diseased conditions as most studies generally examine a disease state versus matched control. Therefore, it is important to further address how demographics such as age, race, and sex impact EV levels. For example, there are few longitudinal studies that examine EVs over time in the same participants. Limited data suggests that healthy individuals retain consistent EV concentration and protein levels over time 32. However, further studies are needed with repeated measures and longer durations between times to make solid conclusions. As observed in Tables 1-3, EV studies report inconsistent information about age, race, and sex of cohort participants and very few address race and sex. This information is vital to making comparisons between studies. With anticipated demographic shifts in the US favoring a larger aging population, it will be important to consider age as a covariate in EV studies. Furthermore, as the majority of EV studies have been conducted using EVs from White individuals, it is also highly imperative to include diverse cohorts and include both males and females.

**Figure 5:**
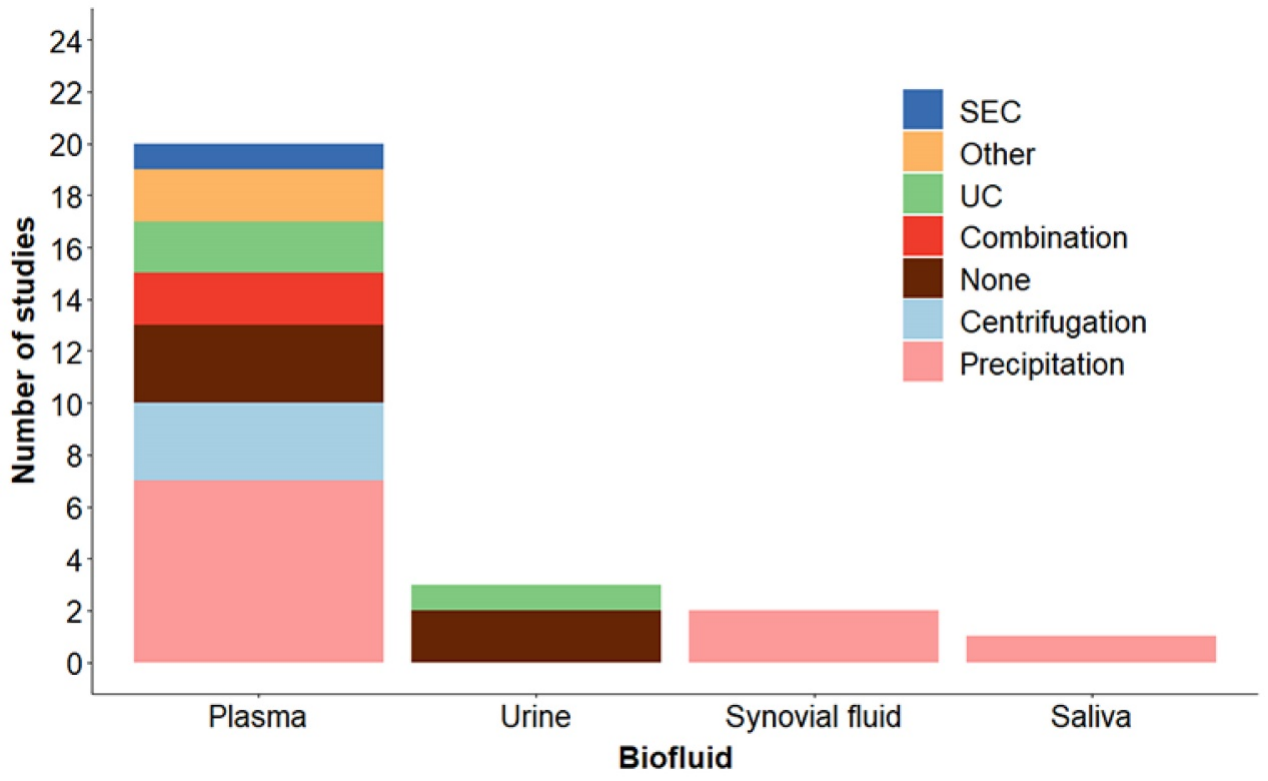
** Methods used for EV separation by biofluid.** Human cohort studies listed in Tables 1, 2 and 3 were compiled by EV separation method and categorized based on biofluid. Details of the methods are described in the Tables and listed as: Precipitation methods (Precipitation), low speed centrifugation (Centrifugation), ultracentrifugation (UC), Combination (two techniques were combined), Size exclusion chromatography (SEC), None (no EV separation, EVs were detected by flow cytometry), and Other (see Tables for details).

It remains difficult to determine the appropriate technique to utilize for EV separation from biofluids. In the field there is no consensus on the “best method” for EV separation from biofluids and MISEV states that in general “There is no single optimal separation method, so choose based on the downstream applications and scientific question.” 12. Separation of EVs to utilize clinically as biomarkers may require a different isolation technique than other EV studies. However, this is not informative in helping researchers choose the best method for their particular application. We compiled the separation methods utilized in studies discussed here by biofluid. These results are presented in Figure 5. As shown, the EV separation methods vary by biofluid. Precipitation methods are the most utilized, most likely due to the limited sample volumes required, the ease of use, reproducibility and lack of specialized equipment needed. Procedures requiring minimal processing including low speed centrifugation or no separation (EVs are detected using flow cytometry) are also used. Each method has advantages and disadvantages as far as specificity and recovery and these should be considered when determining which method to utilize. Improvements in methodologies for separating EVs, such as methodologies with high purity and efficiency from low biofluid bulk volume is crucial to bridge the EV field with clinical application.

Although there is still much to learn about EVs, the existing data indicates that EVs are promising candidates for diagnostics, prognostics, and therapeutics. As the field expands to greater numbers of EV studies from human samples, we will get a clearer picture of the major influencers on EV characteristics.

## Figures and Tables

**Table 1:** Human cohort studies examining EVs with respect to age.

EV characteristics	Findings	Cohort	Age^1^	Race/ethnicity^2^	Clinical condition	Biofluid	EV isolation method	Ref.
Concentration, Size	↓ EV concentration with ageNo difference in EV size with age	**n = 74; M = 39, F = 35**		AA W	Healthy	Plasma	ExoQuick precip.	32*
Cargo (Protein)	↓ EV levels of apoptosis proteins (p53, cleaved PARP and cleaved Caspase-3) with age↑ EV levels of CD151 and MUCIN16 with age	n = 30M = 16, F = 14	30-35					
		n = 30 M = 17, F = 13	40-55					
Functional effect	EVs from older individuals are internalized more in B cells and activate monocytes compared to EVs from younger individuals	n = 14 M = 6, F = 8	55-64					
Concentration, Size	No differences in EV concentration or size with age	**n = 35; M = 18, F = 17**		ASN W Mé	Healthy	Plasma	Iodixanol density gradientsize-exclusion chromatography	35
Cargo (Protein)	EV protein differences between age groups	n = 12 M = 5, F = 7	20-39					
Functional effect	EVs from middle and older age groups (>40 years) increase the colony-forming capacity of hematopoietic stem cells compared to untreated and EVs from young subjects	n = 11 M = 7, F = 4	40-59					
		n = 12M = 6, F = 6	60-85					
Concentration	↓ % of CD31+ in SEV, MEV and LEV; CD81+and CD9+ in SEV and MEV; HLA-ABC+ and HLA-DRDPDQ+ in SEV with age	n = 12 M = 6, F = 6	40 (22-58)	Not reported	Healthy	Plasma	None^3^	34
Cargo	↓ % and # CD29+ and CD31+ EVs with active mitochondria with age. ↓ % CD9+ EVs carrying respiring mitochondria with human age. ↓MitoTracker Deep Red MFI with age in multiple EV subpopulations							
Concentration	No difference in EV concentration, total protein concentration, or CD63+ EV levels between young and old	**n = 6; M = 6**		Not reported	Not reported	Plasma	Size-exclusion chromatography	36
		n = 3(M = 3)	25-35					
		n = 3(M = 3)	65-75					
Concentration	↓ in CD41a+, CD105+, and CD235a+ EV_MP_ with age	n = 143M = 80, F = 63	40.4 (25-53)	Not reported	Healthy	Plasma	None^3^	33*
Cargo (Protein)	No difference in lymphocyte markers on EVs with age	**n = 51; M = 22, F = 29**		Not reported	18 Healthy adults 33 Aged individuals	Plasma	Centrifugation(20,000 *g*)	39
		n = 6	20-29					
Functional effect	EVs from different aged donors can affect T cell activation	n = 5	30-39					
		n = 7	40-49					
		n = 6	70-79					
		n = 10	80-89					
		n = 13	90-99					
		n = 4	100-104					
Cargo (DNA (mtDNA))	↓ Plasma EV mtDNA with age	**n = 55 (visit 1), 67 (visit 2)**		AA W	Healthy	Plasma	ExoQuick precip.	40*
Functional effect	Cells treated with EVs from young individuals had significantly higher levels of both basal and maximal respiration compared to cells treated with EVs from old individuals	n = 21; M = 12, F = 9 (visit 1) and n = 25; M = 16, F = 9 (visit 2)	30-35					
		n = 28; M = 17, F = 11 (visit 1) and n = 29; M = 17, F = 12 (visit 2)	40-55					
		n = 6; M = 1, F = 5 (visit 1) and n = 13; M = 5, F = 8 (visit 2)	55-64					
Concentration	↓ CD63+,β-1 AR+ and nephrin+ uEVs with age	n = 138M = 69, F = 69	20-70	Not reported	Kidney donors	Urine	None^3^	38
Cargo (miRNA)	↑ miR-24-3p in EVs from older individuals.	**n = 28; M = 14, F = 14**		Not reported	Healthy	Saliva	Total exosome isolation kit (Invitrogen)	48*
		n = 15; M = 8, F = 7	21-26					
		n = 13; M = 6, F = 7	61.5 - 72.5					

^1^Age reported as mean and/or age range. ^2^Race abbreviations are as follows: AA = African American, ASN = Asian, HSP = Hispanic, Mé = Métis, W = White. ^3^No EV isolation method; detection through flow cytometry *Covariates were included in statistical analyses. % = percentage; # = number; EV = extracellular vesicles; F = females; LEVs = large EVs; M = males; MEVs = mediums EVs; precip.= precipitation; SEVs = small EVs; Tech. = Technologies; uEVs = urinary EVs

**Table 2:** Human cohort studies examining EVs with respect to race.

EV characteristics	Findings	Cohort	Age^1^	Race/ethnicity^2^	Clinical condition	Biofluid	EV isolation method	Ref.
Concentration, Size	No race differences in EV concentration or size	n = 100M = 50, F = 50	40-55	AA W	Healthy	Plasma	ExoQuick precip.	58*
Cargo(Protein)	↑ levels of phospho-p53, total p53, cleaved Caspase 3, ERK 1/2 and phospho-AKT in W compared to AA							
Concentration	↑ EV concentration in prostate cancer v. healthy in both AA and W	Not reported	Not reported	AA W	Prostate cancer	Plasma	Ultracentrifugation	60
Size	EV size consistent between AA and W between cancer and healthy participants							
Cargo(Protein)	AA and W prostate cancer patients share 57 EV proteins6 proteins in EVs found exclusively in AA (CC2D1A, CCDC172, UPF0728 protein C10orf53, Immunoglobulin kappa variable 1-5 (both region EU and region Cum), DNAJC13)42 proteins in EVs found exclusively in W							
Concentration	No racial differences in EV concentration	**n = 74; M = 39, F = 35**		AA W	Healthy	Plasma	ExoQuick precip.	32*
		n = 30AA = 14, W = 16	30-35					
		n = 30 AA = 15, W = 15	40-55					
		n = 14 AA = 9, W = 5	55-64					
Cargo(Protein)	↑ EV levels of XIAP, c-IAP-2 and Survivin in AA v. W patients with prostate cancer	n = 82Non-cancer M = 10Prostate Cancer M = 72(W = 31 M, AA = 41 M)	48-87	AA W	Prostate cancer	Plasma and serum	ExoQuick precip.	61
Cargo(Protein)	Comparison of proteins in EVs that overlapped or were unique in AA, W, and HSP prostate cancer patients. 22 unique proteins in EVs in AA, 13 in W, and 78 in HSP with prostate cancer	n = 21Non-cancer M = 9Prostate Cancer M = 12 (W = 4, AA = 4, HSP = 4)	Non-cancer = 38.0 (26-45)W = 62.5 (48 - 73)AA = 58.1 (48-75)HSP = 66.0 (65-68)	AA W HSP	Prostate cancer	Plasma	ExoQuick precip.	62
Cargo(Protein)	↑ in EV levels of annexin A2 in AA TNBC-F v. W TNBC-F	n = 167Non-cancer F = 58(AA = 29, W = 27)Breast cancer F = 109 (AA = 41, W = 63)	Not reported	AA W	TNBC	Plasma	Total exosome isolation reagent (Life Tech.)	63

^1^Age reported as mean and/or age range. ^2^Race abbreviations are as follows: AA = African American, HSP = Hispanic, W = White. *Covariates were included in statistical analyses. EV = extracellular vesicles; F = females; M = males; precip.= precipitation; Tech. = Technologies; TNBC = triple negative breast cancer.

**Table 3:** Human cohort studies examining EVs with respect to sex.

EV characteristics	Findings	Cohort	Age^1^	Race/ethnicity^2^	Clinical condition	Biofluid	EV isolation method	Ref.
Concentration, Size	No difference in concentration or size in F v. M	n = 100M = 50, F = 50	40-55	AA = 50W = 50	Healthy	Plasma	ExoQuick precip.	58*
Cargo(Protein)	↑ phospho-IGF-1R levels in F v. M							
Concentration and Size	No sex differences in concentration and size	n = 35M = 18, F = 17	20-85	ASN, W Mé	Healthy	Plasma	Iodixanol density gradient size-exclusion chromatography	35
Concentration	↓ CD62E+ EV_MP_ after moderate-intensity continuous exercise v. baseline in F, but not in M ↑ CD62E+ EV_MP_ levels after high-intensity interval exercise v. moderate-intensity continuous exercise in M.	n = 20M = 10, F = 10	18-40	Not reported	Exercise intensity	Plasma	None^3^	68
Concentration	↓ erythrocyte and stem cell progenitor EV_MP_ and ↑ in endothelial EV_MP_ in pre-menopausal F v. M↑ erythrocyte-derived EV_MP_ in post-menopausal F v pre-menopausal F↑ PS+ and P-selectin+ EV_MP_ in F v. M	n = 144M = 62, F = 82	20-70	Not reported	Healthy	Plasma	Centrifugation (20,000 *g*)	66
Concentration	EV levels differed between M and F post-exercise	Obesen = 15 (M = 7, F = 8 )	Obese 21.2 ± 8.8)	Not reported	Acute exercise in obese and normal weight	Plasma	Ultracentrifugation	79*
		Normal-weight n = 8(M = 4, F = 4)	Normal weight (26.2 ± 7.2)					
Concentration	↑ Annexin V+, platelet (CD61+ and P-selectin+) and endothelial (E-selectin+) EV_MP_ in F v. M↑ Annexin V+, platelet (CD63+, CD61+, and P-selectin+) and endothelial (E-selectin+) EV_MP_ in F during luteal phase v. M↑ Annexin V+, CD63+, CD61+ and ↓ E-selectin+ EV_MP_ in F in luteal v. follicular phase of menstrual cycle	n = 45M = 18, F = 27	Not reported	Not reported	Healthy; different phases of menstrual cycle	Plasma	Centrifugation (17,570 *g*)	80
Concentration	Various EV_MP_ levels were similiar between F and M↓ in CD105+ EV_MP_ in F >55 yrs of age compared to <55 yrs	n = 143M = 80, F = 63	40.4 (25.25-53)	Not reported	Healthy	Plasma	None^3^	33
Cargo(Protein,RNA)	No difference reported for protein or RNA							
Cargo(Protein)	Sex differences in neuronal-derived EV protein levels between cognitively normal and cognitively impaired individuals with or without HIV infection	n = 80M = 29, F = 51	Not reported	Not reported	HIV-associated neurocognitive disorders	Plasma	ExoQuick precipitation andimmuno-precip.	69*
Cargo(Protein)	5 proteins (Serpin C1, Serpin G1, Serpin F2, CD14, Cystatin C) in EVs were different in F with and without IHD	Control n = 257 (65% F)Case (with stable IHD)n = 187 (46% F)	67.65 ± 9.10	Not reported	Ischemic heart disease	Plasma	Lipo-protein subfraction isolation	65*
Cargo(Protein)	No EV protein sex differences in overall cohortEV levels of CD171, PD-L1 and CD151 were higher in M v. F smokers↓ EV levels of TSG101, AREG, MUC1, CD146, CD13, CEA, EGFR, CD142, Ny-ESO-1, EpCAM, and PLAP in F smokers v. F non-smokers. ↑ EV levels of HER2, c-MET, CD171, PD-L1, and CD151 in M smokers v. M non-smokers↓ EV levels of AREG and MUC1 with age in M	n = 161M = 90, F = 71(Non-smoking M = 62, Non-smoking F = 48)	40-69	Not reported	Healthy v. smoking status	Plasma	EV array	64
Cargo(miRNA)	↓ EV levels of miR-146a-5p and miR-21-5p in F following ethanol intoxication↑ EV levels of miR-182-5p in M following ethanol intoxication	Control n = 18 (M = 9, F = 9)Neuroinflammation n = 18 (M = 9, F = 9)	M = 20.67F = 19.88	Not reported	Alcohol-induced neuroinflammation	Plasma	Total exosome isolation kit (Invitrogen)	67
Cargo(miRNA)	↑miR-34a in apoptosis-derived EV_EMP_ in M v. F↓ miR-125a in activation-derived EV_EMP_ in M v. F	n = 30M = 15, F = 15	56 ± 6	Not reported	Healthy	Plasma	None^2^	81
Concentration	↓uEVs in F v. M	n = 19 M = 7, F = 12	58 +12	Not reported	Kidney donors	Urine	Differential ultracentrifugation	71
Concentration	↑ uEVs in F v. M↑ PS+, CD63+, SM22 alpha+, and Claudin-1+ in F vs. M	n=138M=69 F=69	20-70	Not reported	Kidney donors	Urine	None^3^	38
Concentration	Differences in uEV populations of various inflammatory and kidney-specific protein markers between both control and kidney stone positive F and M	n = 110M = 60, F = 50	19-76	Not reported	Kidney stone	Urine	None^3^	70
Cargo(miRNA)	↓ 69 miRNAs in OA-M v. non-OA-M↓ 91 miRNAs in OA-F v. non-OA-F↑ 52 miRNAs in OA-F v. non-OA-F	n = 6 non-OA (M = 3, F = 3)n = 8 OA (M = 4, F = 4 )	47.42	Not reported	Osteoarthritis	Synovial fluid	Total exosome isolation kit (Life Tech.)	72
Cargo(Protein)	7 proteins (SAGA-associated factor 29 homolog, COL6A1, Complement component 5 variant, KIAA1466 protein, Beta-2-glycoprotein I, FLJ94908 highly similar to PPAR binding protein,3'-5' exonuclease TREX2 long form) in EVs differ between OA-M v. non-OA-M12 proteins (HP fragment, HP, V1-5 protein fragment, Alpha-1-Acid Glycoprotein 1, Anti-HER3 scFv, APOL1, anti-folate binding protein, C1QC, HRV Fab N27-VL, CP fragment, CP, Myosin-reactive immunoglobulin heavy chain variable region) in EVs differ between OA-F v. non-OA-F	n = 15 non-OA(M = 7, F = 8)n = 17 OA(M = 7, F = 10)	47.42	Not reported	Osteoarthritis	Synovial fluid	Total exosome isolation kit (Life Tech.)	73

^1^Age reported as mean and/or age range. ^2^Race abbreviations are as follows: AA = African American, HSP = Hispanic, Mé = Métis, W = White. *Covariates were included in statistical analyses. EV = extracellular vesicles; EV_MP_ = EVs defined as microparticles or microvesicles; EV_EMP_=endothelial-derived EVs defined as microparticles or microvesicles; F = females; IHD = Ischemic heart disease; M = males; OA = Osteoarthritis; precip.= precipitation; Tech. = Technologies; uEVs = urinary EVs
